# Comparison of Modified Kessler and McLarney Techniques in Zone II Flexor Tendon Repair

**DOI:** 10.7759/cureus.29364

**Published:** 2022-09-20

**Authors:** Hamza Benameur, Souhail Bensaleh, Anis Chagou, Abdeloihab Jaafar, Mohammed CHAHBOUNI

**Affiliations:** 1 Orthopedics and Traumatology, Cheikh Khalifa International University Hospital, Mohammed VI University of Health Sciences (UM6SS), Casablanca, MAR; 2 Orthopedics and Traumatology, Cheikh Khalifa International University Hospital, Mohammed VI University of Health Sciences (UM6SS), casablanca, MAR

**Keywords:** flexor tendon rupture, hand rehabilitation, two-strand technique, four-strand technique, hand injuries

## Abstract

Introduction

Hand injuries are common in the routine practice of any upper limb surgeon. The laceration of the flexor tendons can engage the functional prognosis of the hand. Hence, there exist a multitude pf suturing techniques whose goal is to have a solid repair, allowing an early rehabilitation. Our study aims at comparing the functional results after flexor tendon repairs in zone II using two different techniques, modified Kessler technique and McLarney technique.

Methods

Our study included 42 patients, divided into two groups, one having benefited from the modified Kessler technique and the other from the McLarney technique. The modified Strickland classification was used to compare the functional results at six months after surgery of the two techniques.

Results

Our study showed a better post-operative functional outcome with a lower risk of post-operative rupture in patients operated with the McLarney four-strand technique compared to patients operated with the modified Kessler two-strand technique.

Conclusion

Hand wounds in zone II remain a therapeutic challenge for any orthopedic surgeon due to the multiplicity of factors involved in the prognosis, in particular the type of suture. The suture with more than two strands has proven its effectiveness and its reproducibility, making it possible to find the balance sought by the surgeon, namely a suture that is not cumbersome, easy and quick to perform, and strong enough to start early rehabilitation.

## Introduction

Flexor tendon lacerations represent a challenge for the surgeon, whether for initial care or during follow-up for functional rehabilitation. The area of the hand affected can worsen the functional prognosis, particularly when flexors are lacerated in zone II [1,2]. The tendon repair requires the realization of a main axial stitch, uniting the two ends of section. The number of strands composing the stitch can vary according to the techniques, ranging from two strands up to six strands, as well as the number of knots [3]. This axial stitch is completed by a circumferential epitendinous continuous suture, regularizing the tendon surface, in order to reduce the risk of adherence and increase the chances of healing [4]. The multiplicity of suturing techniques that have emerged over the past few decades highlights the lack of therapeutic consensus. However, the two-strand and four-strand techniques remain the most widely used in current practice. Our study aims at comparing the functional results after repair of the flexor tendons of the fingers in zone II, according to the two techniques.

## Materials and methods

This is a retrospective, comparative study, carried out at the Cheikh Khalifa International University Hospital in Casablanca, Morocco, between June 2018 and June 2022. The study included patients who presented with a unidigital laceration of at least one flexor digitorum profundus tendon in zone II of the hand according to Verdan, and aged over 16 years. We selected patients from two surgeons, one using only the modified Kessler two-strand suture technique and the other using only the McLarney four-strand technique. The type of anesthesia used was either general or locoregional. A pneumatic tourniquet was used for all patients, inflated to 250 mm Hg, for a duration varying between 1 hour and 2 hours depending on the associated lesions. All patients underwent proximal and distal enlargement according to Bruner. The threads used were Prolene 3-0 for the axial stitches and Prolene 6-0 for reinforcement in the anterior hemi-circumferential continuous suture. Pulleys A2 and A4 were preserved. Skin closure in our patients was performed with separate stitches using rapid Vicryl 4-0. A posterior splint with slight flexion of the wrist and fingers in resin, from the forearm to the hand, was made in the operating room for all patients and maintained for 45 days. All our patients benefited from the same rehabilitation protocol based on protected early active mobilization in the same rehabilitation center located in our hospital. The protocol took place in three phases. The first phase that lasted 45 days consisted of a passive and then active mobilization of the fingers, without resistance, protected by a posterior splint, maintaining the wrist and the metacarpal-phalangeal joints in slight flexion and interphalangeal joints in extension. The second phase continued until the third month, with total mobilization of the joints, removal of the splint, and total rolling of the fingers. As for the third phase, it had the objective of muscle strengthening and mobilization against resistance of the fingers. Patients with neglected flexor tendon lacerations were excluded from the study, as well as those with flexor tendon lacerations associated with extensor tendon sections or phalanx fractures, those with flexor tendon lacerations outside zone II, those with multi-digital wounds, those who did not follow the rehabilitation protocol, those who could not be followed up for at least six months. The measure of the mobility of the proximal interphalangeal (PIP) and distal interphalangeal (DIP) joints made it possible to evaluate the functional recovery at six months after the repair according to the adjusted Strickland classification [5] (Table 1), by a physiatrist, without being aware of the technique used.

**Table 1:** Original and adjusted Strickland classification [5] PIP, proximal interphalangeal joint; DIP, distal interphalangeal joint

Score	Original Strickland %	Adjusted Strickland %
Excellent	85-100	75-100
Good	70-84	50-74
Fair	50-69	24-49
Poor	<50	0-24
Strickland = (active flexion PIP + DIP) - (extension deficit PIP + DIP) X 100% / 175°		

## Results

**Figure 1:**
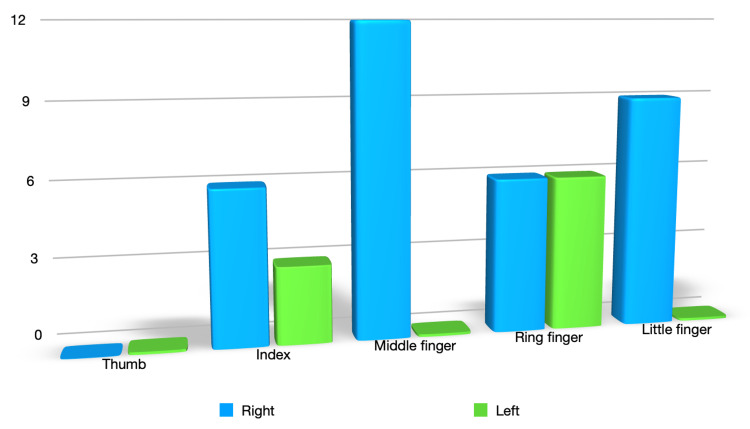
Distribution of tendinous lacerations according to the fingers of the hand

The study included 42 patients, of whom 21 were operated on using the modified Kessler two-strand technique and 21 were operated on using the McLarney four-strand technique. Thirty-three patients presented with lacerations of the flexor digitorum superficialis and profundus, whereas nine patients presented with a section of the flexor digitorum profundus alone. Only 15 flexor digitorum superficialis were repaired due to digital canal congestion (seven in Kessler's patient group and eight in McLarney's patient group). Overall, 78% of wounds concerned the right hand (33 right hands against 9 left hands). The distribution of tendinous lacerations according to the fingers of the hand is shown in Figure 1.

Of our patients, 85.7% were right-handed (36 right-handed patients and 6 left-handed). Fifteen patients presented with laceration of the pedicles. The average age was 32 years (range: 18-66 years), and 64% of patients were male. Injuries were primarily due to armed robbery using a knife (57% of cases), accidents at work (21.5% of cases), and domestic accidents (21.5% of cases); 90% of Kessler's patient group and 88% of McLarney's patient group underwent surgery the same day.

The measurements of degrees of flexion of the PIP and DIP and their extension deficit, at six months after surgery, have been reported in Tables 2, 3.

**Table 2:** The degrees of flexion and extension deficits of the PIP and DIP of the patients operated by the two-strand technique reported to the Strickland classification. PIP, proximal interphalangeal joint; DIP, distal interphalangeal joint

	Flexion		Extension deficit		Strickland
	PIP	DIP	PIP	DIP	
Patient 1	85	63	15	5	73.14%
Patient 2	80	35	10	5	57%
Patient 3	84	42	10	6	63%
Patient 4	91	38	12	5	64%
Patient 5	90	45	10	5	68.57%
Patient 6	68	37	10	10	48.57%
Patient 7	57	29	13	9	36.57%
Patient 8	84	63	13	5	73.71%
Patient 9	80	42	12	7	58.85%
Patient 10	90	40	13	5	64%
Patient 11	85	62	15	5	72.57%
Patient 12	86	53	11	7	69.14%
Patient 13	82	32	9	7	56%
Patient 14	86	47	12	6	65.71%
Patient 15	83	45	14	5	62.28%
Patient 16	55	38	11	6	43.42%
Patient 17	45	38	10	5	38.85%
Patient 18	43	39	11	6	37.14%
Patient 19	45	37	10	6	37.71%
Patient 20	80	60	12	8	68.57%
Patient 21	70	40	11	5	53.71%

**Table 3:** The degrees of flexion and extension deficits of the PIP and DIP of the patients operated by the four-strand technique reported to the Strickland classification. PIP, proximal interphalangeal joint; DIP, distal interphalangeal joint

	Flexion		Extension deficit		Strickland
	PIP	DIP	PIP	DIP	
Patient 22	92	75	5	0	92.57%
Patient 23	90	70	10	5	82.85%
Patient 24	95	70	7	7	86.28%
Patient 25	87	75	8	5	85.14%
Patient 26	84	63	12	6	73.71%
Patient 27	84	65	12	8	48.57%
Patient 28	75	55	13	7	62.85%
Patient 29	90	72	10	5	84%
Patient 30	92	70	10	5	84%
Patient 31	89	75	10	5	85.14%
Patient 32	89	74	7	6	85.71%
Patient 33	90	72	11	6	82.85%
Patient 34	85	75	9	5	83.42%
Patient 35	84	75	7	5	84%
Patient 36	89	76	10	6	85.14%
Patient 37	75	67	7	7	73.14%
Patient 38	74	68	7	7	73.14%
Patient 39	78	68	10	10	72%
Patient 40	69	68	8	5	70.85%
Patient 41	68	59	10	8	62.28%
Patient 42	69	60	10	8	63.42%

According to the adjusted Strickland classification, the results were good to excellent in patients who underwent a four-strand suture, while the results were fair to good in patients who underwent a two-strand repair (Table 4).

**Table 4:** Results according to the adjusted Strickland classification of patients operated on by the two-strand and four-strand technique.

Results	Adjusted Strickland classification	
	Two-strand technique	Four-strand technique
Excellent	0	12
Good	15	9
Fair	6	0
Poor	0	0

There were two cases of rupture after repair in patients operated on using the modified Kessler technique, which occurred during the first phase of finger mobilization, whereas there were no cases of rupture in patients operated on using the McLarney technique.

## Discussion

Laceration of the flexor tendons is a frequent reason for consultation in the emergency room [6], often causing a handicap that can delay or even sometimes hinder the reintegration into the workplace. Hand wounds can involve all zones according to Verdan; however, zone II remains the area most prone to bad results [1,2], mainly due to the simultaneous presence, in a narrow space, of the flexor digitorum superficialis and the flexor digitorum profundus. These two tendons are covered by a digital sheath, reinforced by the various pulleys, of which the most important biomechanically are the A2 and the A4 pulleys. Outside this sheath pass two collateral pedicles formed by the medial and lateral digital artery and nerve, located in front of and on either side of the digital sheath [6].

The high number of finger stiffness and iterative rupture of the flexor tendons has prompted surgeons to imagine different suturing techniques. The expected requirement of these techniques is to have stitches thin enough not to encumber the digital canal and at the same time resistant enough to allow early mobilization, without impeding the sliding of the tendons in the digital sheath, while preserving the vascularization of the tendons as much as possible [3,7].

Different sutures have been studied, in vitro as well as in vivo, with different numbers of strands and knots, with the aim of achieving the greatest resistance while promoting the sliding of the tendon in its sheath, during mobilization, throughout the tendon healing period, which is estimated at 12 weeks on average [8-13].

Our study focused on two techniques used in our structure, the two-strand modified Kessler technique and the McLarney four-strand technique.

Kessler and Nissim's original two-strand technique was introduced in 1969. It consisted of framing the tendon section with a suture thread, with the making of a knot at each angle of the suture [14]. Several surgeons were inspired by Kessler's technique, such as Kirchmayr, Urbaniak, and Pennington, who made certain modifications, such as making loops instead of knots in the corners of the stitch, passing the transverse segment of the thread superficially relative to the vertical segment, and the realization of the final knot in the edge of section [15].

McLarney's four-strand technique was described in 1999, combining two parallel strands and two strands that cross in the section, made with the same suture thread in successive steps, providing high strength, with a time of realization of the suture comparable to that of Kessler [13].

Several studies have emerged comparing two-strand and four-strand sutures over the past few years in patients with wounds in different areas or on multiple fingers [8-12,16]. These studies demonstrated the superiority of more than two strands, which would promote early mobilization with less risk of rupture during the critical period of tendon healing.

In our study, and for the sake of uniqueness, we only included single-digital wounds, unlike other studies, to limit as much as possible the factors that could interfere with our results. These results were judged according to the modified Strickland classification [5]. The purpose of this classification is to assess the post-operative functional recovery of fingers, without taking into consideration the metacarpal-phalangeal joint.

The Strickland classification was created in 1980 [17]. Unlike the TAM (total active motion), it does not take into consideration the metacarpophalangeal joint. Due to its strictness in its original form, Strickland revised the measurement references of its classification downward, which gave rise to the new version of the Strickland classification in 1985 [18].

Our results showed a slight superiority in patients operated on using the four-strand technique compared to those operated on using the two-strand technique, which agrees with the literature. This finding can be explained by the high resistance of the four-strand sutures compared to that of the two-strand sutures, allowing early mobilization with a low risk of postoperative rupture [8-12,16].

After tendon repair, the chosen rehabilitation protocol should promote early mobilization. There is no unanimity on the type of protocol, but the recommended principles are early active mobilization, avoiding adhesions, and promoting joint flexibility [19-21].

Post-operative immobilization is recommended by all authors after repair of the flexor tendons. This immobilization can be done using resin splints or thermoformed splints [20]. In our study, we used resin splints made in the operating room, which the patient wore during the 45 days of immobilization.

The limitations of our study may lie in the fact that our patients were operated on by two different surgeons. We did not measure grip strength due to the lack of a dynamometer in our structure. The realization of a study with more cases operated by the same surgeon according to the two techniques could provide more objective results.

## Conclusions

In this study, four-strand sutures demonstrated their superiority over two-strand sutures, both functionally and in terms of the risk of post-operative rupture. Hand wounds in zone II remain a therapeutic challenge for any orthopedic surgeon due to the multiplicity of factors involved in the prognosis, in particular the type of suture, immobilization and its duration, and rehabilitation and its precocity, without forgetting the technicality of the surgeon, who aims to repair these lesions in the most atraumatic way possible. The suture with more than two strands has proven its effectiveness and its reproducibility, making it possible to find the balance sought by the surgeon, namely a suture that is not cumbersome, easy and quick to perform, and strong enough to start early rehabilitation.
